# Perceptions of Advocacy in High School Students: A Pilot Study

**DOI:** 10.7759/cureus.40581

**Published:** 2023-06-17

**Authors:** Meer S Hossain, Etta Conteh, Samina Ismail, Priscilla Francois, Diane Tran, Tracy MacIntosh

**Affiliations:** 1 Medical School, University of Central Florida College of Medicine, Orlando, USA; 2 Clinical Sciences, University of Central Florida College of Medicine, Orlando, USA

**Keywords:** adolescent, medical education, social media effect, high school students, advocacy education, health advocacy

## Abstract

Assessing perceptions and attitudes of advocacy in adolescent populations is an important area of research. Previous studies have shown that advocacy programs in high schools are well-received and help promote health advocacy. This pilot study took place at the University of Central Florida College of Medicine Health Leaders Summer Academy hosted by medical students of the Student National Medical Association. A one-hour interactive workshop was administered to high school students interested in the healthcare field. Pre- and post-survey data were collected to assess participants’ perceptions, methods, and barriers to engaging in advocacy. A total of 29 students were included in this study. Results indicated that students’ definitions of advocacy changed after completing the workshop, as a higher percentage of students indicated that they practiced advocacy (pre-survey, 82.76% versus post-survey, 95.45%). There was a statistically significant difference in perceptions of the importance of advocacy in the student’s future career (pre-survey, 3.82 versus post-survey, 4.15, p = .035). Social media was the most effective and common form of advocacy used (post-survey 72.73%). The most common barrier to practicing advocacy was a lack of education on a particular topic (31.82% post-survey). Overall, the workshop increased participants' interest in engaging in advocacy. Future directions include expanding the study to a larger population sample throughout the Orlando community and researching the use of social media as a tool for advocacy.

## Introduction

The year 2020 brought on an increased awareness and accountability for racial disparities and injustices in the US where adolescent advocates were up front and center. In 2020, the U.S. Census Bureau reported that children from ethnic/racial minorities comprised half of the population aged < 18 years old [1]. As the youth are on the frontlines of social and cultural change, it is imperative that they have the knowledge on how to become an effective advocate for themselves and their communities. According to Merriam-Webster’s dictionary, advocacy is defined as “the act or process of supporting a cause or proposal” [2]. Health advocacy is specifically the process by which individuals or communities attempt to bring about social, environmental, and/or organizational change on behalf of a health goal, program, interest, or population [3]. Some of the foundational concepts of health advocacy include improving social determinants of health and reducing health inequities [4]. Social determinants of health are the conditions in which people live, learn, work, play, and worship [5]. Some examples include access to safe housing, transportation, job opportunities, nutritious food, language, and literacy skills. Negative social determinants such as lack of access to quality education, unhealthy housing, unfavorable work conditions, and neighborhood violence can have severe neurodevelopmental and biological consequences [6]. However, current interventions often focus on individual behavioral modifications related to disease pathology without addressing underlying social determinants of health. A study by Thornton et al. showed that policy interventions that focus on social determinants of health, including interventions targeted at education and early childhood, urban planning, and community development, housing, income enhancement and employment have been the most effective in reducing health disparities [6]. Specifically early childhood interventions have demonstrated effectiveness in improving long-term health outcomes for disadvantaged children and families [6]. Therefore, it is imperative to advocate for such policies to improve community health and well-being. Health advocacy is most often carried out using social media, direct lobbying, protests and demonstrations, and community mobilization.

Previous programs assessing individual and community health advocacy such as tobacco cessation, diabetes prevention, and healthy food access among adolescents have demonstrated an improvement in healthier lifestyles among those studied [7]. However, these community programs did not address the underlying perceptions and barriers to practicing advocacy. Developing educational approaches to health advocacy in high schools presents a unique opportunity to reach many adolescents to promote changes as individuals, schools, and communities. While there are few studies examining high school health advocacy programs, an integrated health advocacy program studied by Kratze et al. was positively received by adolescents and was an effective way for them to become engaged in advocacy and promote health [7]. A study that examined a year-long health advocacy intervention among teenagers that included training and skill-building at the community and state levels, showed an improvement in attitudes pertaining to advocacy initiatives [3]. Graduate programs specific to public health where many hours can be put into advocacy and public policy training have been shown to be highly effective; however, it is important to incorporate advocacy skills and training in K-12 and college, especially for those who do not attend graduate school [3]. Promoting health advocacy is becoming an increasingly important topic of interest at the high school, college, graduate school, and post-graduate levels [3, 7-10]. Promoting health advocacy at a young age has the potential to gear students to practice advocacy in their future careers and training.

Physicians and medical professionals have a duty to promote health advocacy in their communities. Physicians can specifically contribute by providing evidence-based research to develop policies to promote health and advocate for health-related policies, regulations, rules, and laws [11]. Engaging the next generation through mentorship is another form of advocacy that can lead to a profound change in the community. However, previous literature shows that many health educators do not prioritize advocacy in their professional careers. They participate in voting individually, but few engage in higher-level advocacy efforts [3]. Some health educators are reluctant to practice advocacy. Some concerns based on previous studies include not knowing enough, not knowing how to get involved, not feeling like it makes a difference, lack of understanding of workplace policies and fear of repercussions, and lack of time to engage [12]. This too indicates the importance of early advocacy training among students interested in the healthcare field.

Due to the limited knowledge of perceptions of advocacy among high school students, as well as the increased importance of advocacy training among young professionals, we created an advocacy workshop for high school students, hosted during the annual high school healthcare pathway summer program. This study aims to assess knowledge, attitudes, and practices around advocacy: how high school students define advocacy, where they practice advocacy, how important is it in their future career, barriers to performing advocacy, and methods most used to perform advocacy. Understanding the barriers and predictors to advocacy involvement can be used to improve training and increase the involvement of students, and later health professionals in health advocacy and policy efforts.

## Materials and methods

Medical students from the University of Central Florida College of Medicine in Orlando, Florida have opportunities to engage with the surrounding community. One of these opportunities is the College of Medicine Health Leaders Summer Academy (HLSA), a one-week annual summer program that provides educational experiences to high school students interested in pursuing a career in healthcare. The program focuses on students who attend low-income high schools in Osceola, Orange, and Seminole counties in Florida. This program consists of various workshops, presentations, and simulations. The Student National Medical Association (SNMA), a medical student organization committed to supporting current and future underrepresented minority medical students and addressing the needs of underserved communities, hosted a one-hour-long workshop during this summer program in July 2021. The primary focus of this workshop was educating on the basic principles of advocacy.

Participants were screened using inclusion and exclusion criteria. Inclusion criteria were: students must be in high school; students must attend the advocacy workshop and stay for the whole presentation. Exclusion criteria were: students not in high school. Individuals that attended the presentation and fit the inclusion criteria were included in the final study. The screening was done verbally. Parental and minor consent were both obtained for those who chose to participate. University of Central Florida IRB approval (IRB ID: STUDY00003108) was obtained prior to initiating the study. 

Data was extracted from the Qualtrics survey database and is presented as descriptive statistics. Pre-workshop and post-workshop survey results were compared using paired sample t-test on the Statistical Package for Social Sciences (SPSS) version 27.0 (IBM Corp. Armonk, NY) program. Statistical significance was set to p <0.05.

Prior to this workshop led by SNMA, there had not been a workshop similar in nature administered at HLSA. This pilot study was used to assess what high school students know about advocacy and what their primary means of advocacy are. Results from this study can be later applied to future sessions to better meet the needs of this population. Presentation scenarios were borrowed directly from the Tulane University School of Public Health Blog post “Why Healthcare Advocacy is Important” [13].

Due to COVID-19, the 2021 HLSA was held entirely on Zoom, an online communications platform that allows users to connect via video, audio, phone, and chat. Participants were recruited to the program through flyer distributions at their schools. This session was called “What’s Up With Advocacy?” and had a total of 45 participants signed up for the session.

Prior to the session, a pre-survey was administered. The survey was distributed through Qualtrics. This survey consisted of 34 questions including demographic information. Questions were administered as multiple choice, select all that apply, and Likert scale.

The presentation consisted of a didactic portion and an interactive portion where students were randomly assigned to four breakout rooms on Zoom. An outline of the didactic session is included in Appendix 1.

The interactive portion consisted of three patient scenarios where participants would have to advocate for a patient in need. In these scenarios, participants took on the role of a health advocate which can be a family member, friend, volunteer, or a hired professional who can help individuals understand their illnesses and get access to resources. The scenarios were common ethical situations that occur in the clinical setting. The medical student facilitator read out the scenarios. Participants were then encouraged to speak or write in the Zoom chat what they would do in each scenario. After the discussion, the facilitator read the scenario responses. The breakout rooms ended, and general comments were shared at the end of the session. 

The three question scenarios and responses are presented in Appendix 2.

The participants were prompted to complete the post-survey after the workshop. Four additional questions were added to the post-survey compared to the pre-survey.

## Results

Demographic data

Out of the 45 participants who attended the workshop, a total of 29 high school students completed the workshop and both the pre- and post-survey. Therefore, the results of 29 participants were analyzed. Demographic information, presented in Table 1, indicates that a majority of the students were Hispanic (32.35%), followed by Black (26.47%), Asian (23.53%), White (11.76%), and Other (2.94%). Most of the students identified as female (72.41%). The mean age was 15.3 years, with senior and sophomore high schoolers being the most represented, followed by juniors, then freshman.

**Table 1:** Sociodemographic Characteristics of Participants M = mean; SD = standard deviation

Sociodemographic Characteristics of Participants		
Sample Characteristics	%	M (SD)
Gender		
Female	72.41	
Male	20.69	
Other/Prefer not to answer	6.90	
Race		
White	11.76	
Hispanic or Latino	32.35	
Black	26.47	
Asian/Pacific Islander	23.53	
Native American	2.94	
Prefer not to answer	2.94	
Age		15.30 (0.84)
Year in School		
9th	10.34	
10th	31.03	
11th	27.59	
12th	31.03	
Note. N = 29		

Role of advocacy in students’ lives

As presented in Table 2, after completing the workshop, a higher percentage of students indicated that they practiced advocacy, with 82.76% indicating that they did so in the pre-survey versus 95.45% in the post-survey. Most students indicated that they practiced advocacy at school, as well as online and at extracurriculars/clubs/sports. When asked how often the people around them practiced advocacy, a majority of the students chose “sometimes” in both the pre- and post-survey. When asked how important advocacy was in their future or desired career on a scale of 1-5 (with 1 = not important and 5 = extremely important), there was a significant increase in the mean in the pre-survey (mean = 3.82) versus the post-survey (4.15), (p = .035).

**Table 2:** Pre-workshop and Post-workshop Survey Questions and Answers ^a^Slide to choose (1 = not important, 2 = slightly important, 3 = somewhat important, 4 = very important, 5 = extremely important) ^b^Check all that apply ^c^Slide to choose (1 = not helpful, 2 = slightly helpful, 3 = somewhat helpful, 4 = very helpful, 5 = extremely helpful) ^d^Slide to choose (1 = less likely, 2 = equally likely, 3 = more likely) *p <0.05 M = mean; SD = standard deviation

Pre-workshop and Post-workshop Survey Questions and Answers				
Survey Questions	Pre-survey		Post-survey	
	%	M (SD)	%	M (SD)
What is your definition of advocacy?				
Public support for a policy	20.69		31.82	
An individual or group that aims to influence decisions within political, economic, and social institutions	44.83		45.45	
The act of arguing in favor of/supporting something	34.48		22.73	
Have you ever practiced advocacy?				
Yes	82.76		95.45	
No	17.24		4.55	
Where have you practiced advocacy?				
School/in a classroom	39.29		40.91	
Extracurricular/Club/Sport	21.43		18.18	
At home	14.29		4.55	
In a public setting (restaurant, hospital, etc.)	7.14		9.09	
Online	17.86		27.27	
How often do those around you (parents, family, friends, etc.) practice advocacy?				
Not at all	6.90		4.55	
Sometimes	79.31		77.27	
Often	13.79		18.18	
How important is advocacy in your future or desired career? ^a^		3.82*(0.93)		4.15*(0.73)
What is your current primary form of advocacy?				
Social media	35.71		50.00	
Word of mouth	57.14		50.00	
Attending protests	0.00		0.00	
Other	7.14		0.00	
Which social media platforms do you use regularly? (use for at least 5 hours a week) ^b^				
Instagram	45.83		50.00	
Facebook	4.17		5.26	
Twitter	2.08		0.00	
Snapchat	16.67		10.53	
TikTok	31.25		31.58	
Other	0.00		2.63	
What do you think is the most effective form of advocacy?				
Social media	44.83		72.73	
Word of mouth	31.03		9.09	
Attending protests	6.90		0.00	
Legislative action/Lobbying	13.79		9.09	
Other	3.45		9.09	
What do you think are the most influential barriers to your own performance of advocacy?				
Lack of support	6.90		9.09	
Law	3.45		4.55	
Lack of motivation	10.34		13.64	
Willingness to commit time and energy	13.79		4.55	
Limited communication	6.90		0.00	
Lack of education on a particular topic	17.24		31.82	
Fear of backlash	20.69		18.18	
Lack of time	17.24		18.18	
Lack of money	0.00		0.00	
Other	3.45		0.00	
How helpful was this workshop? ^c^				4.27 (0.75)
Do you feel more inclined to participate in advocacy after this workshop? ^d^				2.64 (0.48)
Note. N = 29				

Most common forms of advocacy used by students

Before the workshop, 44.83% of respondents felt that social media was the most effective form of advocacy for students. After the workshop, even more students felt this way (72.73%). In the post-survey, half of the students indicated that social media was their primary form of advocacy (50.00%), as well as word of mouth (50.00%). Nearly half of the students indicated that Instagram was their most regularly used social media platform, followed by TikTok.

Perceived barriers to advocacy

There were many barriers students felt that prevented them from engaging in advocacy. The most common responses included a lack of education on a particular topic (31.82%), fear of backlash (18.18%), and lack of time (18.18%).

Participant feedback

Most participants found this workshop helpful with a mean of 4.27 on a scale of 1-5 (1 = not helpful, 5 = extremely helpful). Many participants also indicated being inclined to participate in advocacy after this workshop with a mean of 2.64 on a scale of 1-3 (1 = less likely, 3 = more likely). Qualitative data from participants for future improvement was also assessed. In the qualitative section, participants indicated wanting more interactive components, more scenarios, more exploration of topics on advocacy beyond the healthcare scope, and specific information about ways that they could become involved in the community. Many responses indicated a high interest in performing advocacy and participating in similar workshops in the future.

## Discussion

Currently, there is limited literature on adolescents and their perceptions of advocacy. This is a preliminary study that seeks to address gaps in the literature on health advocacy in the adolescent and young adult populations.

Based on the results of this study, participants showed an improvement in their understanding of the definition of advocacy and the broad scope that it can entail. Participants also experienced a significant increase in perceived importance of advocacy in their future or desired career as seen by the increase in mean score in the results section. As many of the participants plan to have careers in the medical field, we believe engaging them in an interactive workshop about health advocacy specifically, motivated these students to consider how they could pursue advocacy in their careers. Participating in a session led by medical students who have started their professional careers and are passionate about advocacy, may have also influenced these adolescents to consider how they could integrate advocacy early in their careers. This indicates the importance of mentorship at all levels of medical training. Many health educators are reluctant to engage in advocacy [12]. Engaging adolescents who are interested in pursuing careers in medicine, and planting the seed of advocacy early on, can help create physicians who feel empowered to pursue advocacy in their careers.

Our results indicated that social media was the primary means of advocacy used by our participants. Social media is a fast and convenient way to communicate thoughts and ideas. It has been shown that the internet has had a positive impact on the activities of social advocacy groups by increasing the speed, reach, and effectiveness of communication and order [14]. A literature review performed by Jackson et al. indicated that there are currently few studies exploring social media usage on promoting advocacy and that this is an emerging field of research [15]. An analysis by Jonathan et al. on advocacy groups in the US and their usage of social media as a tool for engagement suggested that it is highly used and preferred by these groups [14]. Of the social media avenues studied (Facebook, Twitter, YouTube, LinkedIn, blogs, SMS, and e-mail) Facebook and Twitter were rated the highest for both usage and active engagement from the community [14]. Our study evaluated the use of social media avenues such as Facebook, Twitter, Instagram, TikTok, and Snapchat. We found that Instagram and TikTok were the most used by our participants. The study by Jonathan et al. was published in 2012 and did not specify an age range studied so that may indicate possible reasons for differences [14]. Our results may indicate the preferred social media methods used by adolescents today. Our results also indicated that the majority of individuals preferred to advocate through social media rather than attend protests in person, signaling a possible shift in advocacy methods. These results and limited past research on social media usage and advocacy, highlight a gap in the literature and a need for further research in this area.

The primary barrier to practicing advocacy in our study was a lack of knowledge. This further indicates the importance of workshops and training sessions that address knowledge. Both quantitative and qualitative feedback indicated high motivation and interest in learning about and performing advocacy. We hope to continue annual advocacy workshops with improvements from our survey results.

Limitations to this study include population sampling, small sample size, and being conducted virtually due to COVID-19. The participants primarily being Hispanic, Black, and Asian is consistent with our goal of supporting underserved and historically marginalized communities in central Florida counties. The results are therefore primarily the perceptions of adolescents from these communities. We hope to expand our study to a general group of adolescents in the future. However, the results are still highly important, as it is imperative that minoritized groups are engaged in advocacy and learn to advocate for the needs of their communities.

Another limitation of this study includes having a small, convenience sample size of 29 participants. This was also due to a limited method of recruitment. We hope to apply what is learned from this study to larger populations.

This session was held entirely virtually on Zoom due to the COVID-19 pandemic. Although the session was well received through Zoom, we believe further in-person workshops would allow for a more interactive experience.

To address the limitations of our study, we hope to gain a larger sample size and increase diversity in the participant sampling to better understand adolescent perceptions. We would also like to extend the idea of creating similar workshops to target undergraduate-level students and medical students. We ultimately hope to create an advocacy curriculum that can be used throughout our school.

## Conclusions

Advocacy training and awareness in adolescent populations is an important area of research that currently has limited data. This information is important as adolescents, specifically those from minoritized groups, need to know how to advocate for themselves and their communities. Our study indicated that advocacy training increased knowledge on the broad definition of advocacy and how it can be used in one’s future career in medicine. It also assessed the primary forms of engagement used by youth, social media. As demonstrated in this small pilot study, small methods of training can have a positive impact on perceptions and attitudes.

## Appendices

Appendix 1

**Table 3:** Didactic Session Outline

Didactic Session Outline
1. A brief introduction to the Student National Medical Association (SNMA) team members and the mission of SNMA
2. Defining advocacy and the types of advocacy (administrative, legal, community)
3. Exploring approaches to advocacy such as bottom-up and top-down models
4. Defining advocacy within communities and how to organize a community
5. Different avenues for practicing advocacy (e.g.: speaking to lawmakers face-to-face, calls, emails and letters, rallies, letters to the editor, etc.)
6. Defining healthcare advocacy and how it can be applied to oneself and others

Appendix 2

**Table 4:** Interactive Case Scenarios and Responses

Interactive Case Scenarios and Responses
Scenario 1: Patient Faces Challenges to Medication Management Prior to the COVID-19 Pandemic: A 75-year-old woman with a heart condition attended events at a local community center and had an active social life. Her activities and routines helped her keep up with her prescriptions. She remembered to go to the pharmacy when she went to choir practice; her afternoon visits with friends reminded her to take her evening meds. However, with the breakdown of her routines and growing isolation, she has started to lose control of her prescription management.
Response: The patient advocate arranges a conference call with the client and her doctors. After confirming the client’s medications and dosages, the advocate researches local pharmacies and advises her client about their hours and delivery options. She arranges for her client to stock up on medicines that may take longer to refill due to the pandemic. From there, the advocate regularly checks in with her client, offering medication reminders and asking about any other needs.
Scenario 2: A Cancer Patient Prefers No Further Hospital Stays: A 35-year-old patient with cancer who has a history of long hospital stays does not want to return to the hospital, despite his worsening health. His family wants to honor his wish but feels ill-equipped to care for him at home.
Response: After making a home visit and conducting a health assessment, the advocate arranges for a hospice care agency to visit the patient’s home for immediate intervention. Next, the advocate organizes 24-hour caregivers and helps the family manage his medication. In this way, the patient can live out his final days as he wishes, and his family has needed support so they can focus on saying goodbye to their loved one.
Scenario 3: A Patient Deals with Conflicting Mammogram Feedback: A month after a routine mammogram, a 40-year-old woman receives a call from the facility that took the scans. They tell her she needs additional exams but offer no clear answer as to why. She makes another appointment, but it is canceled due to equipment malfunction. A week later she arrives for an additional mammogram. After her scans, she receives confusing information at the facility and leaves frustrated. That evening the facility calls and informs her she has a cyst and should follow up in 6 months for repeat imaging. An hour later, the doctor calls informing her she needs another specialist to examine the cyst. The next day, another representative from the facility tells her she needs to see a surgeon.
Response: At the client’s request, the advocate accompanies her to the next mammogram appointment. The advocate then researches specialists in her client’s insurance network and helps her schedule an appointment for a biopsy and a second opinion. Next, the advocate works with the client to coordinate a medical team and treatment plan.
Note. Scenarios are directly from the Tulane University School of Public Health blog “Why Healthcare Advocacy is Important” [13]
